# Individualized counselling for active aging: protocol of a single-blinded, randomized controlled trial among older people (the AGNES intervention study)

**DOI:** 10.1186/s12877-018-1012-z

**Published:** 2019-01-07

**Authors:** Taina Rantanen, Katja Pynnönen, Milla Saajanaho, Sini Siltanen, Laura Karavirta, Katja Kokko, Anu Karvonen, Markku Kauppinen, Timo Rantalainen, Merja Rantakokko, Erja Portegijs, Mary Hassandra

**Affiliations:** Gerontology Research Center, Faculty of Sport and Health Sciences, University of Jyväskylä, P.O. Box 35 (L335), 40014 Jyväskylä, Finland

**Keywords:** Aging, Individualized counselling, Autonomy support, Behavior change, Theory-based intervention, Participation, Mobility, Physical activity, Quality of life

## Abstract

**Background:**

Active aging has been established as a policy goal for aging societies. We define active aging at the individual level as striving for elements of well-being through activities in relation to a person’s goals, functional capacities and opportunities. Increasing evidence suggests that any meaningful activity is beneficial for different aspects of well-being in older people. The aim of the present randomized controlled trial is to test the feasibility and effectiveness of a one-year community-based intervention on active aging. The AGNES intervention aims at increasing older peoples’ participation in self-selected valued activities.

**Methods:**

The proposed study is a two-arm single-blinded randomized controlled trial. The intervention group receives individually tailored counselling for an active life (one face-to-face session, four phone calls and supportive written material) and the control group written general health information only. Two hundred older adults aged 75- and 80- year old, with intermediate mobility function and without cognitive impairment, living independently in the municipality of Jyväskylä, Finland, are recruited and randomized with a 1:1 allocation to the intervention and control group. Randomization is computer-generated stratified by sex and age. The primary outcome is active aging and secondary outcomes are well-being, depressive symptoms, quality of life, personal goals, mobility and physical activity. Measures are administered at pre-trial, mid-trial (at 6 months) and post-trial (12 months after baseline).

**Discussion:**

The AGNES intervention study will provide new knowledge on the effects of individualized counselling on active aging and the potential of older people to promote their own well-being.

**Trial registration:**

The trial is registered at ISRCTN - ISRCTN16172390: Promoting well-being through active aging.

## Background

Active aging is a widely accepted policy goal in Europe and other countries experiencing population aging. The European Union ranks countries according to the Active Aging Index (AAI; [1]). The AAI assesses how well the potential of older people is realized in different member states in line with the prevailing policy agendas, focusing on adding active and healthy years to life. The World Health Organization (WHO) defined the goal of active aging policy in 2002 as follows: “Active aging is the process of optimizing opportunities for health, participation and security in order to enhance quality of life as people age”. The same document also states that “these policies and programs should be based on the rights, needs, preferences and capacities of older people” [2]. This definition emphasizes health, but it also places considerable value on opportunities to participate in meaningful activities according to the individual’s life situation.

In addition to the macro and meso levels discussed above (country, organizational and institutional levels), active aging can also be promoted at the micro level, i.e., in the everyday lives of individual people and families. Many people wish to remain active through engagement in a variety of activities [3], regardless of being in less-than-perfect health. We recently published a definition of active aging that echoes the spirit of the WHO’s macro-level definition but is focused on the individual (micro level): “active aging refers to the striving for elements of well-being through activities relating to a person’s goals, functional capacities and opportunities” [4].

People inherently strive for well-being by pursuing universal needs for competence, autonomy and relatedness [5]. However, the risk for decreased well-being due to passiveness and alienation increases with increasing age, declining functional abilities, (real and perceived) barriers to participation and loss of social ties. Many older people report that preventing passiveness and encouraging participation may add years to life and enhance well-being [6, 7]. Participation in life situations is one of the key dimensions of quality of life in old age [1, 2] and increasing evidence suggests that any meaningful activity is beneficial for different aspects of well-being in older people [8, 9].

We chose individualized counselling as a method for promoting active aging at the micro level, as it provides a feasible and cost-effective way to advance autonomous motivation for self-chosen activities. In the present study, the aim of counselling is to help the participant to increase awareness of meaningful and desirable activities that are likely to yield personal well-being, and to set new self-selected activity goals, foster autonomous motivation and facilitate positive changes in activity.

Earlier counselling studies aiming at increasing activities and participation among older people have mostly concerned participation in physical activity and other health behaviors or have targeted people with specific chronic conditions [10, 11]. Successful interventions have typically incorporated tailored counselling based on theory and personal contact [10–13]. A recent meta-analysis found that health promotion interventions delivered face-to-face and including behavior change techniques such as goal setting, self-monitoring or behavioral practice/rehearsal, or combinations of these, were associated with beneficial changes in multiple motivational constructs [14]*.*

Motivation is a psychological concept defined as ‘a driving force for the goal-directed behavior’ [15]. Influencing motivation in counselling interventions is essential, as it underlies the efforts to change behaviors [14]. Autonomous motivation is linked with positive changes, e.g. in physical activity, and refers to engagement based on pleasure or personal importance to the individual rather than extrinsic control, such as fear of punishment [5]. The integration of two socio-cognitive theoretical models, the self-determination theory [5] and the theory of planned behavior [16], is used as the theoretical framework of the AGNES intervention. The Self-Determination Theory (SDT) is a motivation theory and is concerned with supporting our natural or intrinsic tendencies to behave in effective and healthy ways, whereas the Theory of Planned Behavior (TPB) links one’s beliefs and behavior. A previous meta-analysis provided evidence supporting the integration of the two theories [17], and hence their integration is expected to provide complementary explanations of the processes that underlie motivated behavior. Although the SDT framework has been widely used to explore and explain the motivation behind everyday activities in older people [18–20] and the TPB to explain how older individuals intend to behave [21–25], the integrated model has hitherto only been used as a process model in seeking to explain the behavior of older adults [26]. There is a lack of intervention research testing the integrated model’s ability to explain behavior change in older adults across time.

Our view is that increased activity will also increase well-being. While the dictionary definition of well-being is “a good or satisfactory condition of existence”, in empirical research, well-being is often assessed as the absence of specific indicators of ill-being such as depressive symptoms [27]. Increasing participation in various valued social and leisure activities may alleviate depressive symptoms [28]. Positive indicators of well-being include psychological well-being, which is characterized by, for example, a striving to find one’s true nature, personal growth and autonomy, and purpose in life [29]. However, there is less evidence on how to promote positive aspects of well-being. As our goal is to promote activities in line with a person’s goals and values and to support autonomy, it is likely that achieving this goal will also improve psychological well-being.

The World Health Organization defines quality of life as “the individual’s perception of their position in life in the context of the culture and value systems in which they live and in relation to their goals” [30]. Quality of life is a generic and multidimensional concept, and is often assessed as an outcome in different health enhancing interventions [31]. Due to the breadth of the concept, many different interventions targeting a wide range of activities have been found to improve quality of life among older people [3, 31–33].

Mobility refers to movement in all its forms, such as walking for leisure, daily tasks, activities associated with work and play, exercising, driving a car, and using various forms of public transport. Optimal mobility means the ability to go safely and reliably where one wants to go, when one wants to go, and how one wants to get there [34]. Life-space mobility, the spatial aspect of mobility, is an indicator of access to community amenities and describes a person’s opportunities to participate in meaningful activities outside the home [35, 36]. Consequently, increased participation in meaningful activities will likely increase life-space mobility and physical activity, since moving out-of-home and further away has been associated with higher levels of physical activity among older people [9], regardless of the reason for going out or even the means of transportation.

### Aims

The aim of this study is to develop an individualized counselling intervention that supports older individuals to increase their participation in self-selected valued activities and their involvement in meaningful life-situations and to test its feasibility and effectiveness. Specifically, we will investigate the effects of the intervention on active aging (primary outcome) as well as on well-being, depressive symptoms, quality of life, personal goals, mobility and physical activity (secondary outcomes). Moreover, we will examine the mechanisms underlying participants’ perceived autonomy support and active aging.

## Methods

### Setting

The study is community-based and targets older people whose life-space mobility is intermediate (excluding the most active and most inactive), have no cognitive impairment, and live independently in the municipality of Jyväskylä, Finland. These criteria have been selected as the intervention is expected to best benefit people who have room for improvement in their activity levels and whose physical and mental health allow them to comply with the intervention.

### Trial design

The AGNES intervention is a single-blinded, randomized, controlled, parallel-group, two-arm trial with a 1:1 allocation ratio. The parallel groups are a “Counselling group” (CG), serving as the intervention group, and a “Health information group” (HIG), serving as the control group.

### Blinding

Trained research assistants collecting data at all time- points will be unaware of the group allocation. The statistician performing the analyses will also be blinded to group allocation. After randomization, the participants and the counsellor delivering the intervention will be informed of the group allocation.

### Sequence generation and the allocation concealment mechanism

Stratified randomization for age and sex will be used with a 1:1 allocation to ensure a good balance of participant characteristics in each group. The randomization sequence will be created using Stata 15.0 statistical software (StataCorp, College Station, TX) by the study statistician, who immediately after generation of the random allocation sequence will seal them in envelopes.

### Sample size and power calculations

A total of 168 participants are needed for a 90% probability to detect a treatment difference at a two-sided 0.05 significance level, if the true difference in the main outcome between the intervention and the control group is 10%. As some of the participants may be vulnerable and the intervention is long, 200 participants will be needed to allow for the potential attrition rate of 20% during this time. The power calculations are based on estimates from two earlier studies [37, 38].

### Participants

#### Eligibility criteria

The AGNES intervention will recruit participants from the ongoing AGNES cohort study [39], which is an observational study of three age cohorts (75, 80, and 85 years) living independently in the municipality of Jyväskylä, Finland. Inclusion criteria for the RCT study will be age 75 or 80, a baseline score between 52.3 and 90.0 on the University of Alabama at Birmingham Life-Space Assessment (LSA; [35, 40]), and a score of 25 or higher on the cognitive function test (MMSE; [41]). Recruitment has started in October 2017 and will continue until the number of participants reaches 200.

#### Recruitment process and informed consent

Participants in the AGNES cohort study [39] who have consented to future study requests will be assessed for eligibility for the RCT. Following the cohort study baseline assessments, eligible participants will be offered an opportunity to join the trial. Willing participants will receive a short verbal briefing and written information, including a consent form that they can read at their leisure. Within 2 weeks, the intervention counsellor will call potential participants to confirm their willingness to participate in the study and, if necessary, to provide them with additional information on the study. Once the participant will provided verbal consent, the pre-trial interview will be conducted over the phone. After the interview, the counsellor will open the randomization envelope and inform the participant about the treatment group. The signed written informed consent form will be returned by post or in person.

#### Data collection

**Fig. 1:**
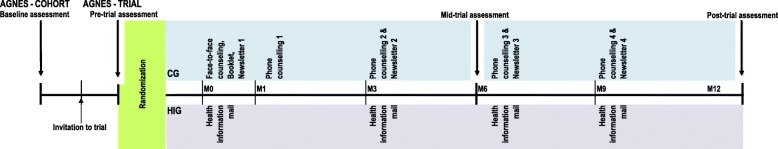
Approximate timeline of data collection in months and participant contacts by group in the AGNES intervention. Participant contacts are indicated with vertical lines

Data collection will be performed at three time points: pre-trial, mid-trial (at sixth months), and post-trial (at 12 months) by trained research assistants. The pre-trial data will derive from a face-to-face interview in the participants’ homes, a postal questionnaire completed by the participant, 1 week of physical activity surveillance using accelerometers and the phone interview conducted by the counsellor. The mid-trial and post-trial data collections will be conducted using face-to-face interviews in the participants’ homes followed by 1 week of physical activity surveillance with accelerometers (post-trial only). The interviewers have participated in a training course on interviewing techniques suitable for use with older people, study ethics and safety as well as practice sessions. Incoming data will be monitored periodically. Figure 1 shows the data collection timeline in months and participant contacts by group.

### Intervention

The active aging counselling intervention targets several different behaviors that vary between individuals. Consequently, we follow the guidelines of the Medical Research Council (MRC) framework for complex interventions [42] and implementation research [43–45]. A central key aim of the AGNES intervention is to support older individuals’ autonomous motivation in seeking to participate in activities that they personally value. Individuals have a key role in setting their goals, planning their actions and monitoring their progress in change process. Another key issue is the individualization of the counselling sessions (face-to-face and phone calls) after profiling the participants, in order to better meet their needs. Profiling is defined as “the recording and analysis of a person’s psychological and behavioral characteristics, so as to assess or predict their capabilities in a certain sphere or to assist in identifying categories of people” [46]. The profiling sources are participants’ baseline data on the main areas of their everyday life such as health, social contacts, well-being, and preferred activities and goals.

#### Counselling group (CG)

After randomization, the intervention (CG) group will participate in a 90-min, face-to-face, individual counselling session on the premises of the University of Jyväskylä. A trained counsellor with previous experience in counselling older adults will deliver all the counselling sessions. The counselling session follows a semi-structured protocol of questions. The main body of the questions initiating discussion topics is the same for all participants. Additional probing questions will be based on the participants’ answers. The main body of questions guiding the counselling session consists of four sections, (a) Introduction and building rapport; b) Current activities and change talk; c) Goal setting, action plans and how to use the supportive materials that we provide (booklet, calendar and newsletter); and d) Wrapping up and closure. During the face-to-face counselling sessions, participants will receive an *information booklet* on active aging [47] from the counsellor. The Information Booklet is 64 pages long and provides information about activities and behaviors that promote active aging, with self-help exercises and techniques that the participants can use to facilitate their planned changes, and a calendar that they can use to monitor their activities. Participants will be instructed to use the information booklet as support during the one-year intervention.

After the initial face-to-face session, participants will receive four phone counselling sessions, at 1, 3, 6 and 9 months after the face-to-face counselling session. The aim of the sessions is to provide them with additional support, feedback and encouragement. The content of the follow-up phone calls will be a discussion about the participant’s progress towards achieving their goals, sharing in their successes or failures, and further encouragement to continue their efforts towards increasing their activities. The duration of these short counselling contacts is expected to be 20–30 min. In addition, participants will receive four printed newsletters, the first during the first counselling session, and the other three by mail 1 week before the phone sessions at 3, 6, and 9 months. The newsletters will feature the activities that are currently organized for older people in the Jyväskylä area and the success stories of people who are living an active life. A description of the Behavior Change Techniques (BCTs) used in the AGNES intervention [48] is displayed in Table 1.

**Table 1:** Behavior change techniques (BCTs^a^) in the AGNES intervention

Group	Behavior change techniques	Face-to-face counselling	Information booklet	Phone counselling	Newsletter
Goals and planning	Goal setting (behavior)	X	X		
	Problem solving	X	X	X	
	Action planning	X	X	X	
	Review behavior goals			X	
	Discrepancy between current behavior and goal			X	
Feedback and monitoring	Self-monitoring of behavior	X	X	X	
Social support	Social support (unspecified)	X		X	
	Social support (practical)	X	X	X	X
	Social support (emotional)	X	X	X	
Natural consequences	Monitoring of emotional consequences	X	X	X	
	Information about health consequences		X		
	Information about social and environmental consequences		X		
	Information about emotional consequences		X		
Identity	Incompatible beliefs	X			
	Valued self-identity	X	X		
Associations	Prompts/cues		X		
Repetition and substitution	Habit formation		X		
	Generalization of a target behavior		X	X	
	Graded tasks		X		
Comparison of outcomes	Credible source		X		X
	Pros and cons		X		
	Comparative imagining of future outcomes		X		
Reward	Self-reward		X	X	
	Social reward			X	
Antecedents	Restructuring the physical environment		X		
	Restructuring the social environment		X		
Comparison of behavior	Social comparison				X

^a^BCTs Version 1

#### Training of the intervention providers

Two intervention providers will receive the training. They both have postgraduate degrees in health sciences and they will take part in six two-hour sessions to develop the skills needed for autonomy-supportive counselling. The training sessions will be supported by printed material and include a group discussion section and a practice session with feedback. The topics of the six sessions were: a) The self-determination approach in behavior change interventions, b) Motivational interviewing strategies, c) Planning autonomy-supportive sessions tailored to the individual, d) Using autonomy-supportive communication/language, e) Applying the behavior change techniques in an autonomy-supportive way, and f) Profiling practice in hypothetical case studies.

#### Control group (health information group)

Participants allocated to the control group will receive printed general health information material of the kind used in usual health care services for older people. The envelope with the printed material (brochures, booklets etc.) will be mailed by post to the control group participants during months 1, 3, 6, and 9. The material is divided into four themes. The first theme is “Exercise”, the second “Nutrition”, the third “Cardiovascular diseases”, and the last “Type II diabetes”. We do not expect the control group to exhibit change in the primary outcome. After completing the post-intervention assessment, the participants of the control group will receive the same information booklet and hence the opportunity to be exposed to the same information as those in the intervention group.

#### AGNES intervention adherence plan

Several strategies for enhancing adherence are integrated into the study plan. For example, frequent contacts (e.g., follow up support, newsletters and health information mails) to decrease attrition and maximize completeness of the data collection; tailoring (e.g., via profiling, readability of printed materials) to increase acceptance and logistical support (e.g., convenient schedule appointments) to limit participant burden. Additionally, an adherence monitoring mechanism will be used to ensure fidelity to the intervention. Research group meetings take place twice per month to monitor the progress of the intervention and to ensure that the protocol plan will be followed. During the first ten face-to-face and five additional randomly selected sessions during the intervention year, the second trained counsellor, using a protocol checklist, will observe the study counsellor in order to give feedback and ensure that the counselling protocol is applied as planned. Moreover, the study counsellor will complete a one-page self-assessment checklist and will make notes after each face-to-face and phone call counselling session. Notes will also be used to feed future follow-up counselling phone calls. Participants who wish to discontinue participation in the study will be asked if they want to remain for the assessments only.

### Outcomes and measures

Table 2 lists all the measures of the study along with references, the data collection methods and time points.

**Table 2:** Measures, data collection time points, methods and references in the AGNES intervention

Assessment / method / scale	Pre-	Mid-	Post-	References
Main outcome				
Active Aging (68 items UJACAS Scale)	HI	HI	HI	[4]
Secondary outcomes				
Psychological well-being (42-item Scale of psychological well-being)	HI	–	HI	[49]
Depressive symptoms (CES-D questionnaire; 20 items)	HI	–	HI	[50]
Quality of life (13-item OPQOL-brief questionnaire)	HI	–	HI	[52]
Personal goals (single item)	PhI	HI	HI	[62]
Life-space mobility (15-item LSA questionnaire)	HI	–	HI	[35]
Walking difficulty	HI	–	HI	[54]
Sense of autonomy outdoor mobility (five-item subscale of IPA)	HI	–	HI	[56]
One-week physical activity surveillance (with tri-axial accelerometers)	HI	–	HI	[58]
Self-reported habitual physical activity (eight-item YAPS)	HI	–	HI	[59]
Theory-based explanatory measures				
Perceived autonomy support (five items)	PhI	HI	HI	[63]
Autonomous-controlled motivation (eight items)	PhI	HI	HI	[64]
Attitude towards active aging (three items); Subjective norms of active aging (single item); Perceived behavioral control over active aging (single item); Intention for active aging (single item)	PhI	HI	HI	[65]
Descriptive measures				
Education (single item) & Perceived financial situation (single item)	HI	–	–	[66]
Physical health	HI	–	–	[67]
Lower extremity physical performance (SPPB)	HI	–	–	[69]
Maximal isometric handgrip strength (hand-held dynamometer)	HI	–	–	[71]
Cognitive functioning (19-item MMSE)	HI	–	–	[41]
Changes in life situation (five items)	PhI	HI	HI	–
Habitation (single item)	HI	–	–	–
Self-rated health (single item)	HI	–	–	[72]
Perceived age (single item)	HI	–	–	[73]
Perceived active aging (single item)	HI	–	–	[4]
Motivation for active aging (single item)	HI	–	–	[4]
Feeling of loneliness (single item)	PQ	–	–	[74]
Social contacts (three items)	PQ	–	–	[67]
Current hobbies (single item)	HI	HI	HI	–
Assessment debriefing (one item)	HI, PhI	HI	HI	–
Intervention debriefing (one item)	–	–	HI	–

For each assessment, according to the time point (pre, mid and post), the method of data collection is indicated as Home Interview (HI), Postal Questionnaire (PQ) or Phone Interview (PhI)

#### Main and secondary outcomes

##### Active aging

The main outcome measure is active aging. Active aging is assessed using the University of Jyväskylä Active Aging Scale (UJACAS) at all time points [4]. The UJACAS consists of 17 items: practicing memory, using a computer, advancing matters in one’s own life, exercising, enjoying the outdoors, taking care of one’s appearance, crafting or DIY, making the home cozy and pleasant, helping others, maintaining friendships, getting to know new people, balancing personal finances, making one’s days interesting, practicing artistic hobbies, participating in events, advancing societal/communal matters, and doing things according to one’s world view. For each item, participants are asked to rate (scale from 0 to 4) the strength of their striving to accomplish the activity, their ability and opportunity to perform the activity and their amount or frequency of doing the activity during the 4 weeks immediately prior to the measurement. Subscores (range 0–68) for the four dimensions of striving, ability, opportunities and activity are then calculated, with higher scores reflecting higher striving, better ability or opportunities, and higher activity. Similarly, a higher composite score (range 0–272) indicates a higher level of active aging. The scale has shown good psychometric properties and test-retest reliability [4].

##### Well-being

*Psychological well-being*

Is assessed using the 42-item version of the Scales of Psychological Well-Being at pre- and post-trial [49]. The 42 items divide into six components, each with seven items. The components are: (a) autonomy (e.g., “My decisions are not usually influenced by what everyone else is doing”), (b) environmental mastery (e.g., “In general, I feel I am in charge of the situation in which I live”), (c) personal growth (e.g., “For me, life has been a continuous process of learning, changing, and growth”), (d) positive relations with others (e.g., “Maintaining close relationships has been difficult and frustrating for me”; reverse scored), (e) purpose in life (e.g., “I have a sense of direction and purpose in life”), and (f) self-acceptance (e.g., “When I look at the story of my life, I am pleased with how things have turned out”). The participant rates the fit of each item on a six-point scale ranging from one (strongly disagree) to six (strongly agree). A total score for the whole scale is computed by summing the scores for the 42 items (range 42–252). Further, a sum score (range 7–42) is calculated for each of the seven components. Higher scores indicate higher psychological well-being.

*Depression*

The 20-item Centre for Epidemiologic Studies Depression Scale (CES-D) [50], is used to assess depressive symptoms at pre- and post-trial. The participant rates the frequency of twenty depressive symptoms during the previous week. The score for each item ranges between zero (rarely or none of the time) and three (most or all of the time). The total score ranges from 0 to 60, with higher scores indicating more depressive symptoms. The reliability and validity of the CES-D scale has been satisfactory [51].

##### Quality of life

Quality of life is assessed pre- and post-trial with a short version of the Quality of Life Questionnaire for Older People (OPQOL-brief). The scale includes 13 items related to life overall, health, participation, social relationships and financial situation. Answers are given on a scale from one (strongly disagree) to five (strongly agree). The sum score ranges from 13 to 65, with higher scores indicating higher quality of life. The scale’s reliability and validity, as a measure of overall quality of life, are satisfactory [52].

*Life-Space Mobility*

Life-space mobility reflects actual mobility performance in daily life and is assessed with the University of Alabama at Birmingham Study of Aging Life-Space Assessment (LSA) at pre- and post-trial [35]. The LSA comprises 15 items and assesses mobility through the different life-space levels (bedroom, other rooms, outside home, neighborhood, town, beyond town), frequency of movement, and need for assistance during the 4 weeks preceding the assessment. The composite score ranges from 0 to 120, higher scores indicating greater life-space mobility. The scale has good test-retest reliability [53].

*Walking difficulty*

Self-reported walking limitation is assessed pre- and post-trial as perceived difficulty in walking 500 m and 2 km [54, 55]. The response range is from one (able to manage without difficulty) to five (unable to manage even with help).

*Autonomy in outdoor mobility*

Perceived sense of autonomy in out-of-home activities, that is, the feeling of having control over the decision to go out whenever, wherever, and however one wants, is assessed pre- and post-trial with the ‘autonomy outdoors’ subscale of the Impact on Participation and Autonomy questionnaire (IPA), which has been validated [56, 57]. Participants rate their perceived opportunities for visiting relatives and friends, making trips and traveling, spending leisure time, meeting other people, and living life the way they want. The sum score ranges from 0 to 20, with higher scores indicating more restrictions on participation.

*Physical activity surveillance*

Willing participants wear a tri-axial accelerometer (13-bit ±16 g, UKK RM42, UKK Terveyspalvelut Oy, Tampere, Finland) continuously for 7 days following the home interview at pre- and post-trial. At post-trial, only participants who have worn the accelerometer at baseline are invited to participate. When wearing the accelerometer, participants keep a short written diary on their daily activities. The accelerometer is set to 100 samples per second. The accelerometer is attached to the anterior aspect of the mid-thigh of the dominant leg and covered with a waterproof self-adhesive film. Movement and non-movement behaviors are analyzed as the number of minutes spent at a particular activity intensity level (e.g. sedentary, light, moderate, and vigorous) [58].

*Self-reported habitual physical activity*

Self-reported habitual physical activity is assessed pre- and post-trial using the Yale Physical Activity Survey for older adults (YAPS; [59]) at the pre- and post-trial home interview. The YAPS questionnaire includes a physical activity dimension sum index, which is the sum of five weighted sub-indices (vigorous physical activity, leisure walking, moving around, standing and sitting) [59]. The total score ranges from 0 to 137, higher scores indicating higher physical activity. The reliability of the survey has been found to be fair [60].

#### Personal goals

Personal goals are asked using an open-ended question based on the Personal Project Analysis [61], but formulated for the purpose of studying older people’s goals [62]. Following a brief introduction on what is meant by personal goals, the participants can report all the goals they have. The total number of goals will be calculated for each participant. Moreover, the goals will be categorized into 25 goals categories and further into seven thematic goal dimensions based on their content [62].

### Theory-based explanatory measures

Additional explanatory data will be collected at baseline, mid- and post-trial, from both groups.

#### Perceived autonomy support

A modified short version of the Health Care Climate Questionnaire [63] is used to assess participants’ perceptions of the degree to which they experience their social environment as autonomy-supportive versus controlling with respect to active aging. Adjustments have been made regarding the context (social support, in general provided by people) and behavior assessed (active aging). The scale consists of five items (e.g.: “I feel that other people around me have provided me with choices and options to live an active life.”) and responses are given on a Likert-type scale ranging from: “1 = strongly disagree” to “7 = strongly agree”. An individual’s score on this scale is the average of his or her responses on the five items.

#### Autonomous-controlled motivation

The Self-Regulation Questionnaire [64] adapted to meet the needs of the intervention, is used to assess individual differences in the types of motivational regulation for being more active. The questionnaire consists of two sub-scales: autonomous motivation (4 items: e.g., “Because I enjoy being an active person”) and controlled motivation (4 items: e.g., “I would feel like a failure if I do not live an active life”). Answers are given on a Likert-type scale ranging from: 1 = “strongly disagree” to 7 = “strongly agree”. The participant’s score for each subscale is the mean score for the 4 items.

The constructs pertaining to the theory of planned behavior have been developed following the recommendations given in the manual for health services researchers [65] for the needs of the present study. *Attitude* towards being more active is assessed with 3 items scored on 7-point semantic differential scales ranging from 1 to 7 (“Aiming to live a more active life during the next six months means to me … pleasant - unpleasant, difficult - easy, useful - useless”). *Subjective norms of active aging* are assessed with one item: “I feel that the people who are important to me would want/expect me to live a more active life for the next six months”. Responses are given on a 7-point scale ranging from 1 = “strongly disagree” to 7 = “strongly agree”. *Perceived behavioral control over active aging* is assessed with one item: “I am confident that I can live a more active life for the next 6 months”. Responses are given on a 7-point scale ranging from 1 = “strongly disagree” to 7 = “strongly agree”. *Intention for active aging* is assessed using one item: “I intend to live a more active life for the next 6 months”. Responses are given on a 7-point scale ranging from: 1 = “strongly disagree” to 7 = “strongly agree”.

### Descriptive measures

#### Additional baseline measures

*Age* and *sex* are drawn from the population register. At baseline, *education* is assessed by number of completed years of education [66]. *Perceived financial situation* is assessed on a five-point scale ranging from one (very good) to five (very poor). *Physical health* is assessed based on self-reported physician-diagnosed diseases. A co-morbidity index similar to one previously used [67] is calculated from a checklist of diseases prompted by 10 categories of chronic diseases and an open-ended question about any other physician-diagnosed chronic conditions. *Lower-extremity physical performance* is assessed by the Short Physical Performance Battery (SPPB) [68–70]. The battery comprises tests on standing balance, walking speed over a 3-m distance, and the ability to rise from a chair. Established cut-off points are used to score each task from zero to four points [69, 70], higher scores indicating better performance. Participants unable to perform a test are assigned the score zero. A sum score is calculated (range 0–12) when at least two tests are completed. *Maximal isometric handgrip strength* is measured on the dominant side with a hand-held adjustable dynamometer (Jamar Plus digital hand dynamometer, Patterson Medical, 6 Cedarburg, WI, USA) and expressed in kg [71]. *Cognitive functioning.* Cognitive functioning is assessed with the Mini-Mental State Examination (MMSE) [41]. The MMSE contains 19 items and the score ranges from 0 to 30. *Changes in life situation.* Major changes in life situation are assessed with five questions on changes during the last 6 months in health status, mobility or functional ability, family situation or other social relationships, hobbies, and other major changes. Answer options are “No changes”, “Yes, positive changes”, “Yes, negative changes” and “Yes, both positive and negative changes”. Those reporting changes are then asked follow-up questions to gain specific information about the changes. Finally, participants are asked what change was most significant for them and what its impact was on their life.

#### Profiling measures

The following baseline measures will be used as additional information to individualize counselling: *Habitation* is categorized into living alone, with a spouse, with children or grandchildren, with siblings, relatives, or other people. *Self-rated health* is asked with the question: “How would you assess the current state of your health in general”? Answers are given on a five-point rating scale from one (very good) to five (very poor) [72]. *Perceived age*, i.e., how people experience their own aging on the personal level, is assessed with the question: “How old do you feel you are?” [73]. *Perceived active aging* is assessed by asking participants to evaluate how active their life is on a scale from 0 (not at all active) to 10 (very active) [4]. *Motivation for active aging* is assessed by asking participants how strongly they agree (1) or disagree (5) with the claim “I have special interests in my life” [4]. *Feeling of loneliness* is assessed with the question: “How often do you feel lonely?” Answers are rated on a four-point scale ranging from very rarely/never to almost always [74]. *Social contacts* are assessed with questions regarding the frequency of contacts with children and other relatives, close friends, and other acquaintances [67]. The response options are daily, weekly, monthly, a few times a year, rarely or not at all, and not having any children or other relatives/friends/acquaintances. *Current hobbies.* An open-ended question, “What are your most important hobbies?” is used to probe participants’ main hobbies. In addition, the measures of personal goals, active aging, well-being, life space mobility and depressive symptoms were used for profiling.

#### Debriefing questions

All interviews will end with an open-ended question asking participants whether they want to share any thoughts and feelings about the interview. Later, after completing the study, participants will be given the opportunity to share any other thoughts and feelings regarding their experiences of participation in the study.

### Data analysis

All analyses will primarily be performed using intention-to-treat analysis and supplemented by per-protocol analysis for all planned outcomes [75]. Means, standard deviations and frequencies will be calculated for continuous variables. Distributions will be tested for normality. The effects of the intervention will be assessed using repeated measures ANOVA and linear mixed models. Generalized estimating equations (GEE) models will be used to analyze differences in changes in discrete variables over the three time points. The level of statistical significance will be set at *p* < 0.05 (two-sided). Full information maximum likelihood (FIML) estimation under the assumption of data missing at random (MAR) will be used to analyze incomplete data. Multiple imputation methods will be used to complete the dataset if necessary. Non-respondent analyses for those who declined to participate will be performed, based on the cohort baseline data. Subgroup analyses for sex, age, socioeconomic status, cognitive (MMSE) and mobility (SPPB) function will also be performed. In all future publications, data will be reported following the criteria recommended by the CONSORT guidelines.

#### Additional analyses

**Fig. 2:**
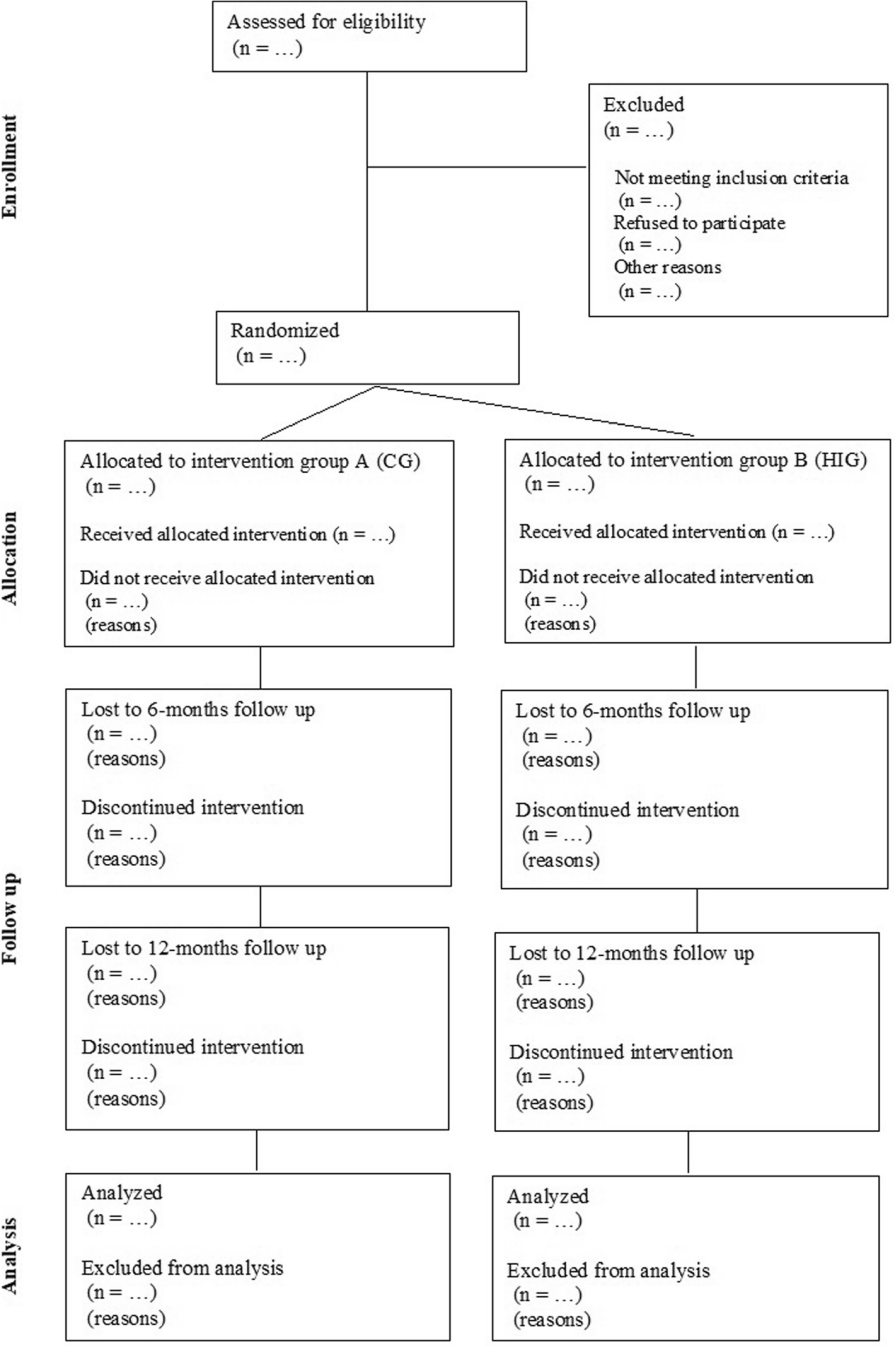
Participant flow diagram in the AGNES intervention

To examine the explanatory mediators, path modelling for mediation analysis will be used. We will test the model at mid- and post- trial with active aging as the primary dependent variable, the intervention condition as the independent variable, and the psychological variables (perceived autonomy support, autonomous motivation, attitudes, subjective norms, perceived behavioral control and intentions) as multiple mediators. Potential trajectories of the outcome and explanatory variables will also be examined. The participant flow diagram during the trial is displayed in Fig. 2.

### Data management and data reporting

The University of Jyväskylä has full ownership of the research data. Consent forms and paper questionnaires will be stored in locked cabinets in a project researcher’s office. Computer assisted interviewing techniques are used, whenever possible. Digital data will be stored on computer drives of the Information Management Center of the University of Jyväskylä. All datasets will be pseudonymized. Data will be managed by AGNES research team members appointed for this task. The key for participant identification will be stored separately from the data files and only accessible to designated research team members. Research team members can use pseudonymized datasets for research and teaching purposes, providing they are employed by the University of Jyväskylä. Results will mainly be reported in articles published in established international scientific journals and presentations in scientific and professional congresses. The researchers will target open access publishing and comply with the University of Jyvaskyla recommendation for parallel publishing in the open access digital JYX repository. Results will be communicated at events and in meetings and through traditional and social media for professionals and the general public.

## Discussion

The AGNES intervention study will provide new knowledge on the effects of individualized counselling on active aging. We will test the idea that supporting autonomous motivation to engage in personally important preferred activities will increase participation in these activities, and eventually also improve different aspects of well-being. Novel features of the AGNES intervention trial include the theory-based counselling intervention and the outcome of active aging, which is quantifiably assessed with a recently launched measure [4].

The idea for the intervention stems from our earlier observational studies and randomized controlled trials. As we have reported previously [76], personal goals correlate with life-space mobility, a variable which indicates the extent to which a person participates in different activities outside the home. Our earlier randomized controlled trial on the effects of physical activity counselling bears similarities with the present study in counselling approach used [77]. The trial proved efficacious in increasing physical activity [78]. Since conducting these studies, additional evidence has accumulated suggesting that any activity outside the home will have benefits induced, e.g., by increased physical activity and more social interaction with others; see, e.g., [8, 9]. We have therefore expanded the scope of the intervention to promote any self-selected activity and refined the counselling approach based on new knowledge on behavior change techniques.

The present study will produce new knowledge on the potential of older people to promote their own well-being. We aim to update knowledge on the positive aspects of aging and activity in older age. We also expect further knowledge and theory on successful counselling methods.

We are currently recruiting participants for the AGNES intervention study from the population-based AGNES cohort study [39], which uses probability sampling. This will likely help us avoid some of the biases characteristic of convenience sampling, i.e., that participants represent individuals who are the most interested in the study topic. We are starting by excluding the most active volunteers in order to recruit participants who have room for improvement. We are also excluding those whose life-space mobility is very low, as it is likely that they experience many barriers to increasing activity on their own initiative and would probably benefit from a more intensive intervention. We are following the guidelines for planning and conducting randomized trials. The research group meets regularly to discuss the progress of the study. The counsellor delivering the intervention has a support system and the counselling sessions are periodically monitored.

The study site is a medium-sized Finnish city (population 139.260) that is age-friendly and provides many activity opportunities for older people. It is likely that different living environments need different study approaches. For example, rural areas and big cities present different opportunities and challenges for participation. Although the intervention will be delivered to all participants assigned to it by a highly motivated and well-trained professional counsellor, the intervention may nonetheless be considered a trial in a real world setting in the sense that the population is community-based and rather heterogeneous, which in turn means that variation in compliance is inevitable.

## Abbreviations

AAI: Active Aging Index
BCT: Behavior Change Techniques
CES-D: Centre for Epidemiologic studies Depression Scale
CG: Counselling group
FIML: Full information maximum likelihood
HI: Home interview
HIG: Health information group
IPA: Impact on Participation and Autonomy questionnaire
LSA: University of Alabama at Birmingham Study of Aging Life-Space Assessment
MAR: Missing at random
MMSE: Mini-Mental State Examination
OPQOL: Older People’s Quality of Life questionnaire
PhI: Phone interview
PQ: Postal questionnaire
SDT: Self-Determination Theory
SPPB: Short Physical Performance Battery
TPB: Theory of Planned Behavior
UJACAS: University of Jyvaskyla Active Aging Scale
